# Supplementary material for: Electron transfer in the respiratory chain at low salinity
Source: Nat Commun. 2024 Sep 19;15:8241. doi: 10.1038/s41467-024-52475-3 (PMC11413003; doi:10.1038/s41467-024-52475-3)
Supplement: Supplementary file 1 — Supplementary information [file 41467_2024_52475_MOESM1_ESM.pdf]

Supporting information

Electron transfer in the respiratory chain at low salinity

Ana Paula Lobez[1,a], Fei Wu[1,a], Justin Di Trani[b], John L. Rubinstein[b,c,d], Mikael Oliveberg[2,a], Peter Brzezinski[2,a] and Agnes Moe[2,a,e]

[a] Department of Biochemistry and Biophysics, The Arrhenius Laboratories for Natural Sciences, Stockholm University, SE-106 91 Stockholm, Sweden.
[b] Molecular Medicine program, The Hospital for Sick Children, 686 Bay Street, Toronto, Ontario, Canada M5G 0A4
[c] Department of Medical Biophysics, The University of Toronto, 101 College Street, Toronto, Ontario, Canada M5G 1L7
[d] Department of Biochemistry, The University of Toronto, 1 Kings College Circle, Toronto, Ontario, Canada M5S 1A8
[e] Present address: Institute of Biochemistry and Molecular Medicine, University of Bern, Bühlstrasse 28, 3012 Bern.

[1] A.P.L. and F.W. contributed equally to this work.

[2]Correspondence: Mikael Oliveberg, mikael@dbb.su.se; Agnes Moe, agnes.moe@dbb.su.se (agnes.moe@unibe.ch); Peter Brzezinski, peterb@dbb.su.se

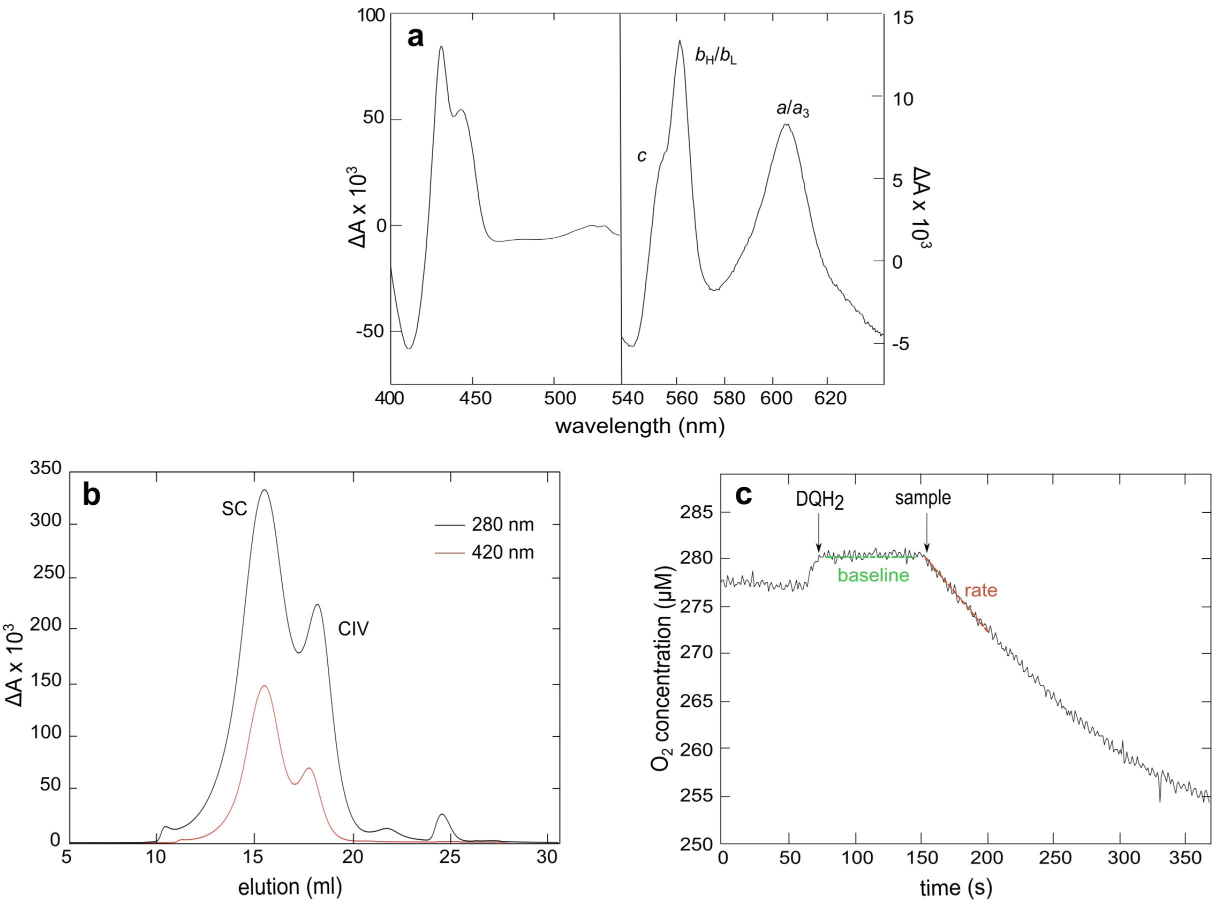

Supplementary Figure S1. Purification and activity of the *S. cerevisiae* supercomplex. (**a**) Reduced-minus-oxidized difference spectrum of the purified supercomplex after size exclusion chromatography in 20 mM KCl, 20 mM KH_2PO_4-KOH (pH 7.4), 0.01 % GDN. The difference absorption coefficients used to calculate the concentration were the following: ε_{554}=21 mM^{-1} cm^{-1} (cyt. *c* and c_1) ε_{562}=51.2 mM^{-1} cm^{-1} (hemes *b*) and ε_{603}=25 mM^{-1}cm^{-1} (heme *a*) [1,2]. (**b**) Size-exclusion chromatography elution profile obtained with a Superose 6 Increase 10/300 GL column in 150 mM KCl, 20 mM KH_2PO_4-KOH (pH 7.4), 0.01 % GDN. Elution was followed at 280 nm (total protein) and at 420 nm (hemes). Reduced-minus oxidized difference spectra of the fractions showed the presence of supercomplex (SC) in the eluted first peak and free CIV in the second peak. (**c**) Quinol oxidation-O_2 reduction rate over time of the *S. cerevisiae* CIII-CIV supercomplex. The turnover rate was calculated from the difference of the slopes after (rate) and before (baseline) addition of the supercomplex.

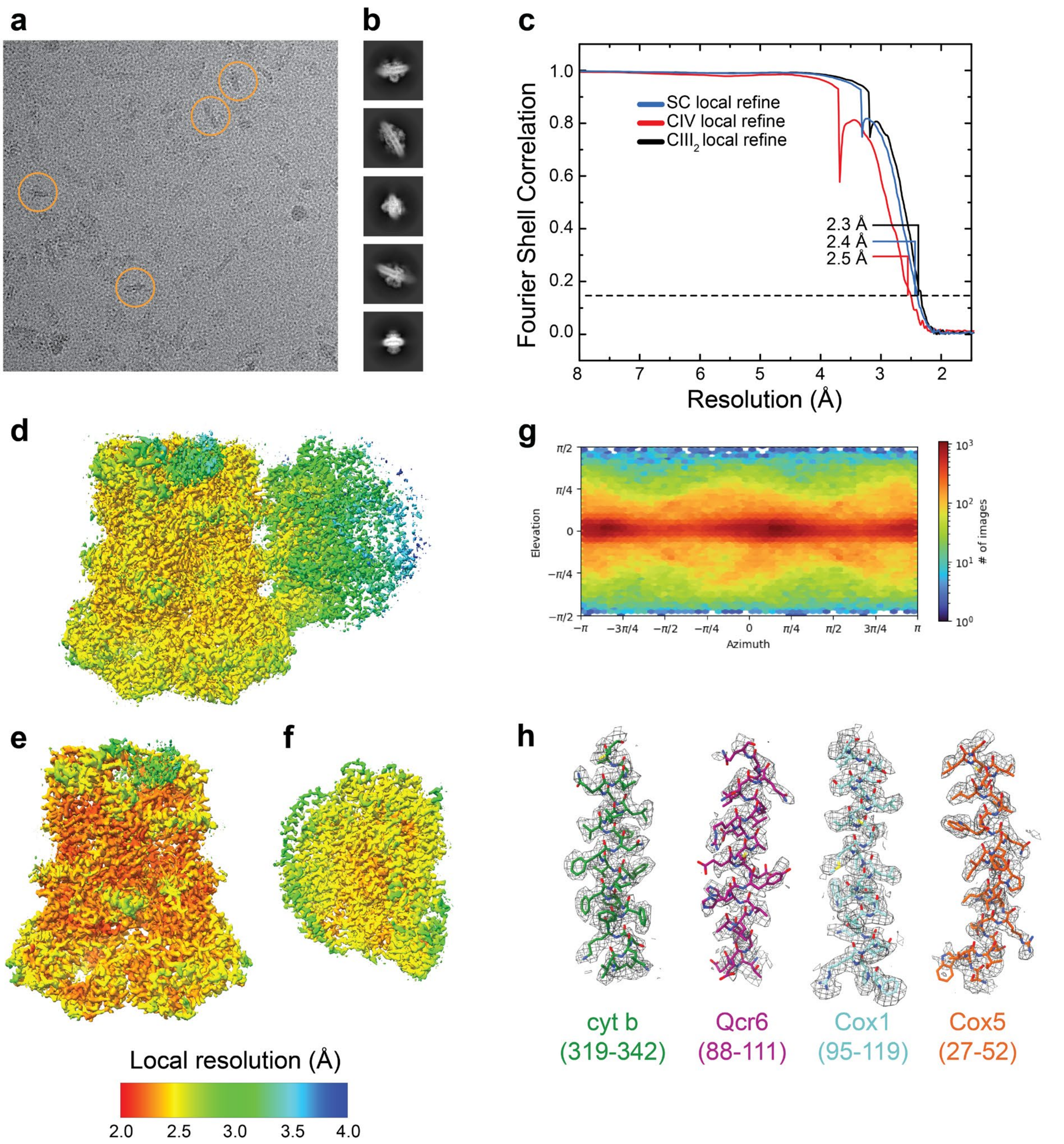

Supplementary Figure S2. Cryo-EM validation. (**a**) Example micrograph. (**b**) 2D class averages. (**c**) Fourier shell correlation (FSC) curves after refinement, corrected for the effects of masking. (**d**) Local resolution of the $CIII_2CIV_1$ map. (**e**) $CIII_2$ local refinement. (**f**) CIV local refinement. (**g**) Viewing direction distribution for particle images. (**h**) Examples of model in map fit.

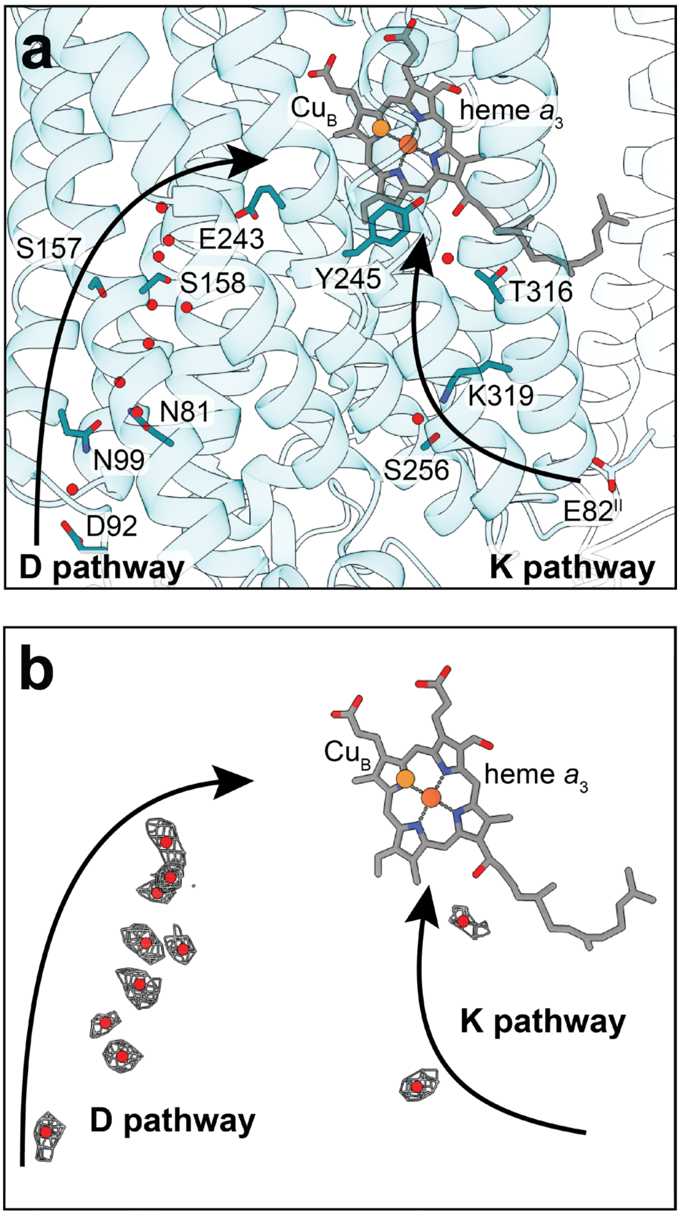

Supplementary Figure S3. Proton pathways. (**a**) Water molecules (red spheres) were resolved in the D and K proton pathways of CIV. The active site and residues of the D and K pathway are indicated. (**b**) Densities of resolved water molecules.

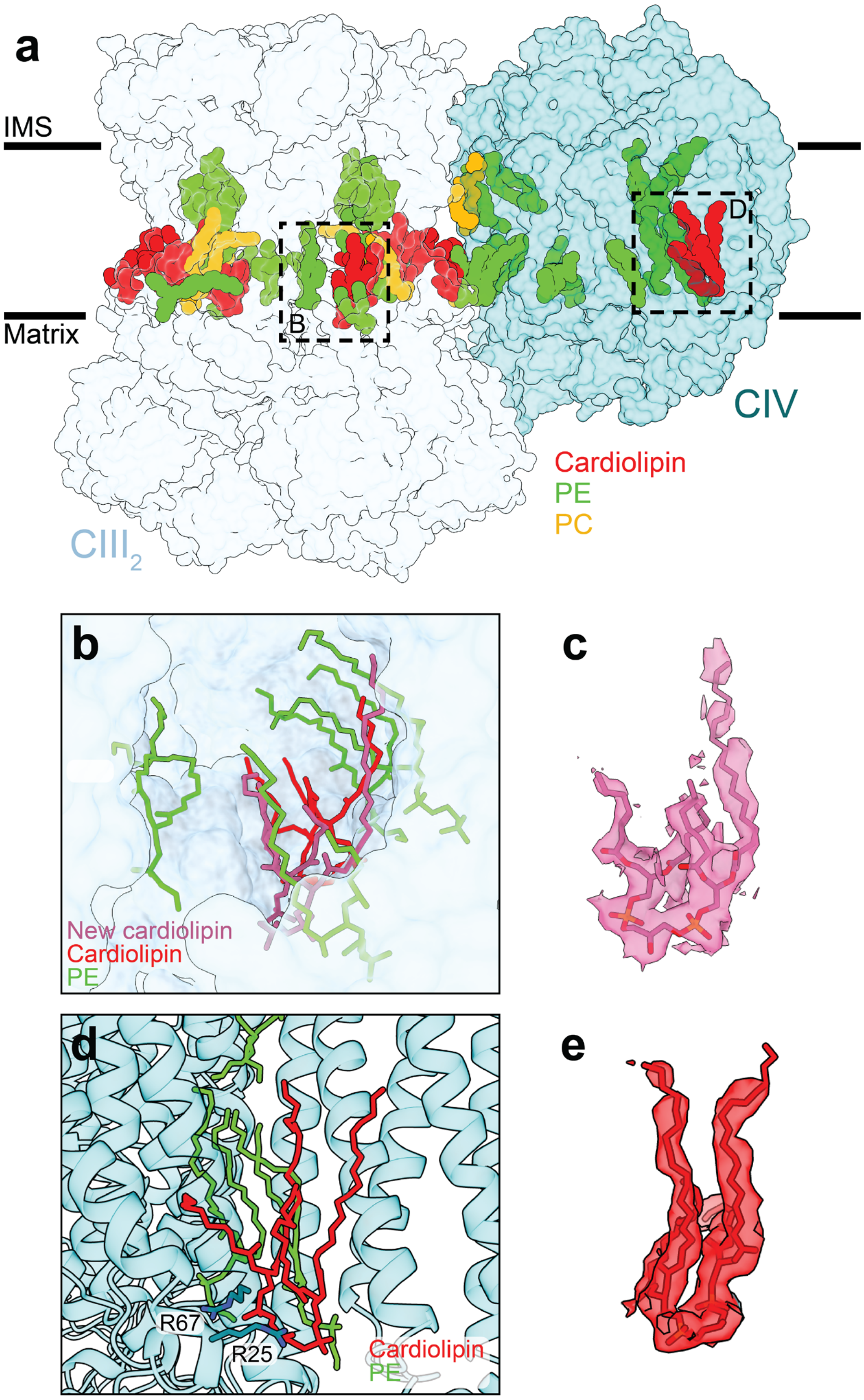

Supplementary Figure S4. Lipids. (**a**) Overview of lipids modelled in the $CIII_2CIV_1$ supercomplex. (**b**) Cardiolipin in a $CIII_2$ cavity, previously modelled as two PE molecules in *S. cerevisiae*. (**c**) Density for the cardiolipin in (b) showing the glycerol backbone connecting the two phosphatidic acid moieties. (**d**) Cardiolipin outside the cavity in CIV, defined by a V-shaped cleft in Cox3, suggested to be used for O_2 diffusion to the CIV catalytic site. Arg67 (Cox3) and Arg25 (Cox7) located close to the cardiolipin molecule are indicated. (**e**) Density for the cardiolipin in (d) showing the glycerol backbone connecting the two phosphatidic acid moieties.

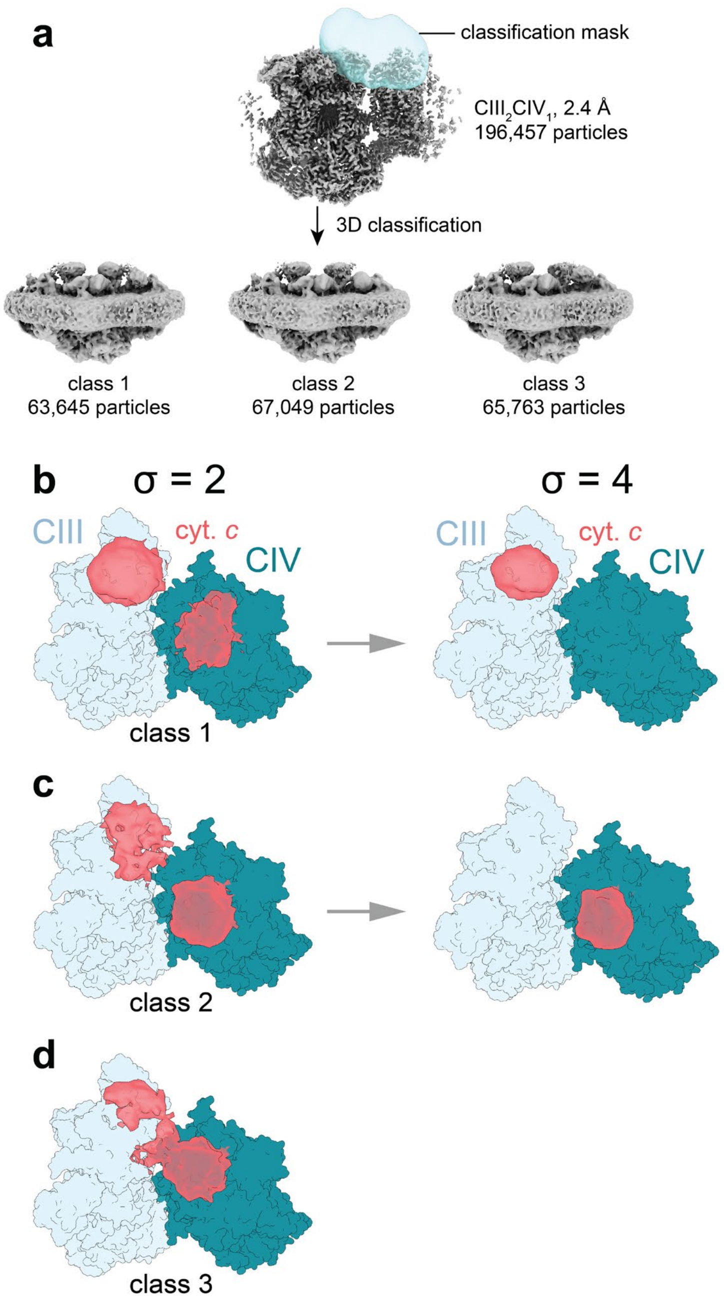

Supplementary Figure S5. Cryo-EM 3D classification. (**a**) $CIII_2CIV_1$ supercomplex with the mask (light blue) and processing pipeline. Particle images could be divided into three classes. (**b**) Class 1 with cyt. *c* bound to a CIII monomer of $CIII_2$ (CIII in the figure) and CIV. The cyt. *c* molecule bound to a CIII monomer (in $CIII_2$) is visible already at σ = 4 while the second cyt. *c* molecule appears at σ = 2. (**c**) Class 2 with cyt. *c* bound to a CIII monomer of $CIII_2$ and CIV. The cyt. *c* molecule bound to CIV is visible already at σ = 4 while the second cyt. *c* molecule appears at σ = 2. (**d**) Class 3 with a diffuse cyt. *c* density in between a CIII monomer of $CIII_2$ and an adjacent CIV, partly overlapping with CIII and CIV.

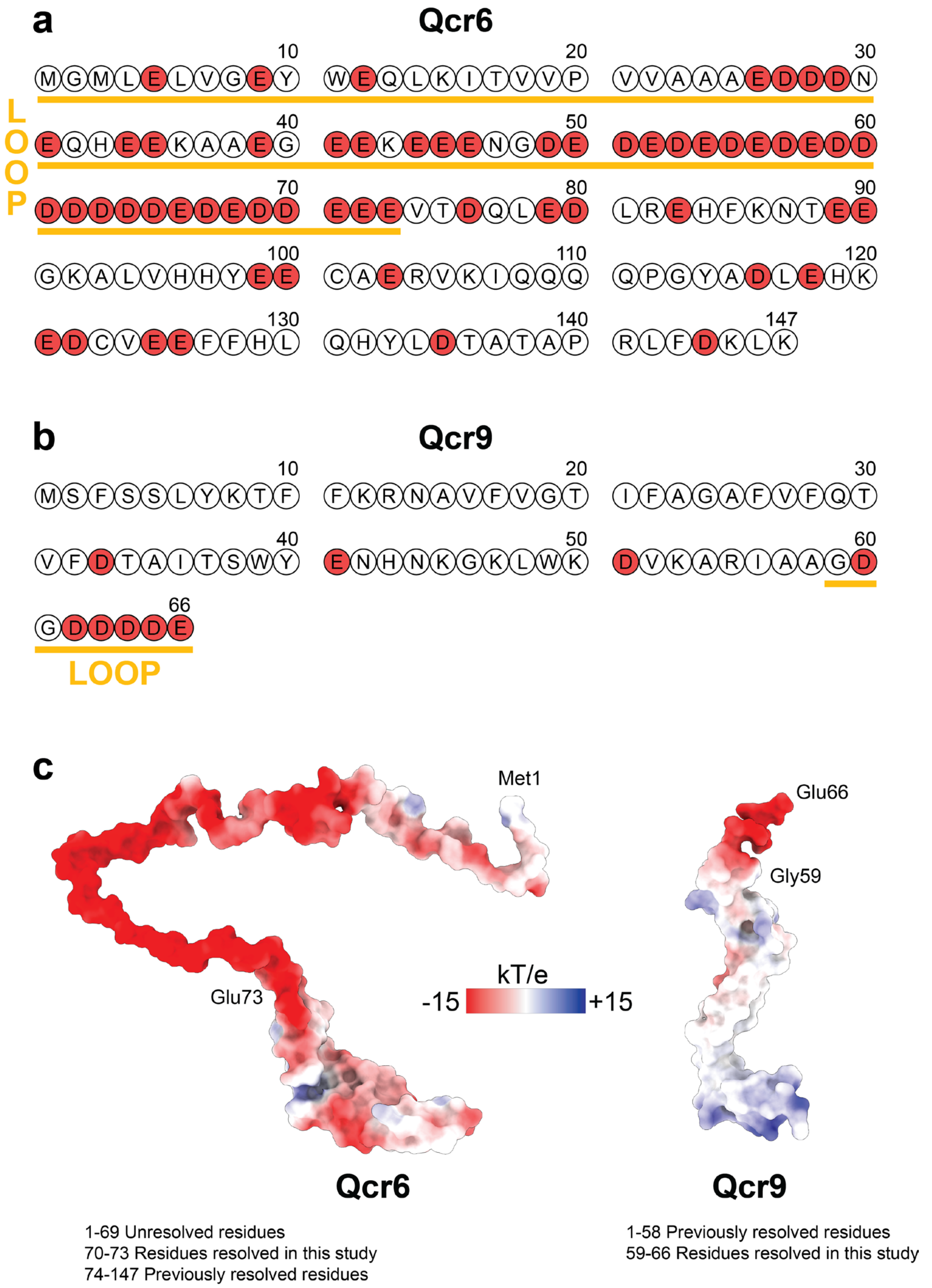

Supplementary Figure S6. Sequence and electrostatic potential of Qcr6 and Qcr9. (**a**) Sequence of Qcr6 from *S. cerevisiae* (Uniprot: P00127) with negatively charged residues indicated in red. The position of the unresolved loop (residues 1-73) is indicated in orange. (**b**) Sequence of Qcr9 from *S. cerevisiae* (Uniprot: P22289) with negatively charged residues indicated in red. The position of the unresolved loop (residues 59-66) is indicated in orange. (**c**) Coulomb electrostatic surface representation of Qcr6 and Qcr9. Predicted models from AlphaFold Protein Structure Database [3] were used to illustrate the two complete subunits.

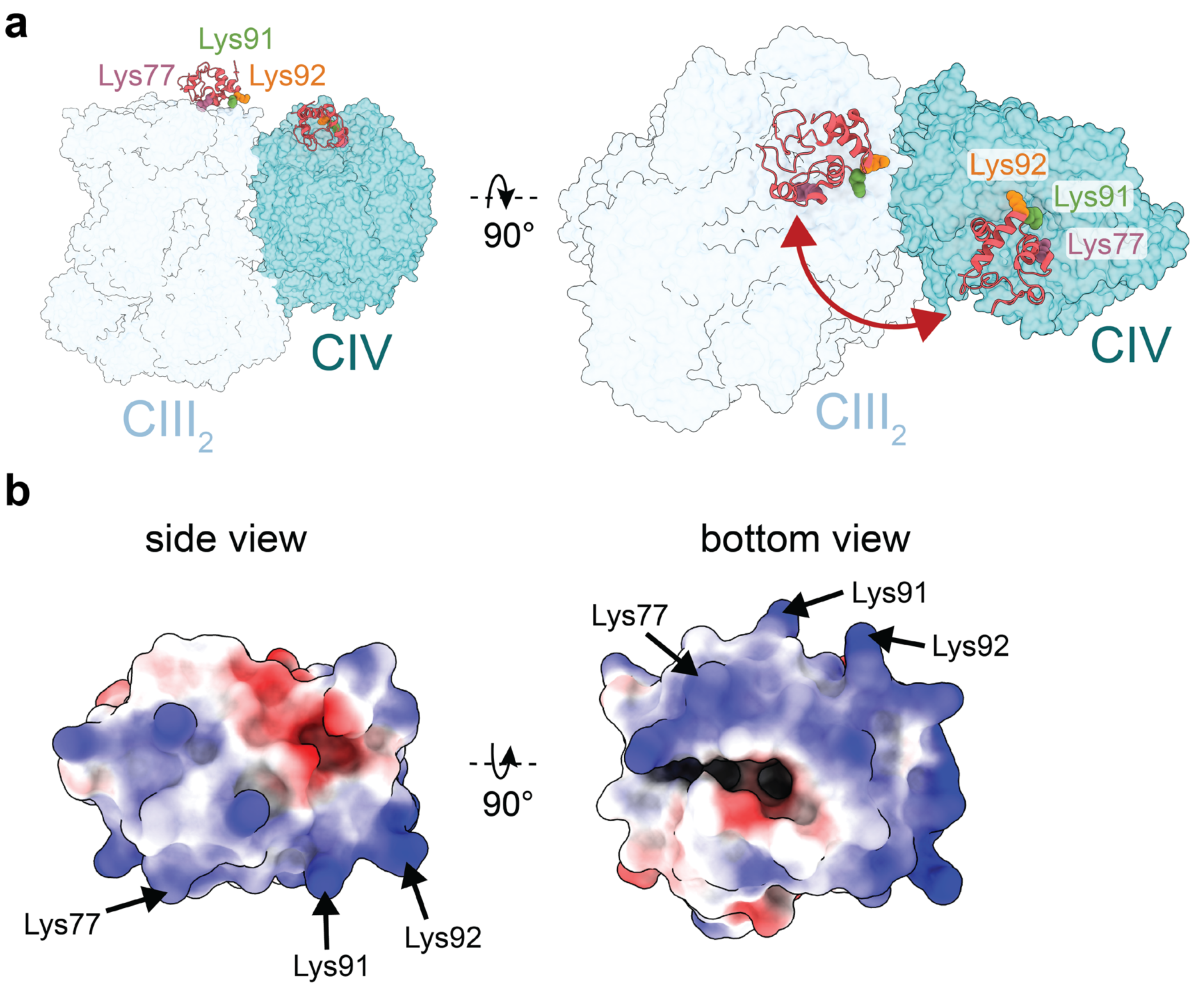

Supplementary Figure S7. Cyt. *c* rotation and electrostatic surface. (**a**) Cyt. *c* bound at CIII and CIV from the crystal structures of the CIII-cyt. *c* [4] (*S. cerevisiae* cyt. *c* and cyt. bc_1) and CIV-cyt. *c* [5] (modelled for *S. cerevisiae* cyt. *c* and CIV, based on the crystal structure of the mammalian counterparts) co-complexes, respectively. Residues Lys77, Lys91 and Lys92 (*S. cerevisiae* cyt. *c* amino-acid residue numbering) are equivalent to Lys72, Lys86 and Lys87 (horse heart cyt. *c* amino-acid residue numbering), previously shown to be involved in binding (but not mediating electron transfer) of cyt. *c* to the bovine Cyt*c*O [6]. (**b**) Coulomb electrostatic potential surface for *S. cerevisiae* cyt. *c* with the three Lys residues involved in cyt. *c* binding to CIV (and presumably also CIII, [4,7]).

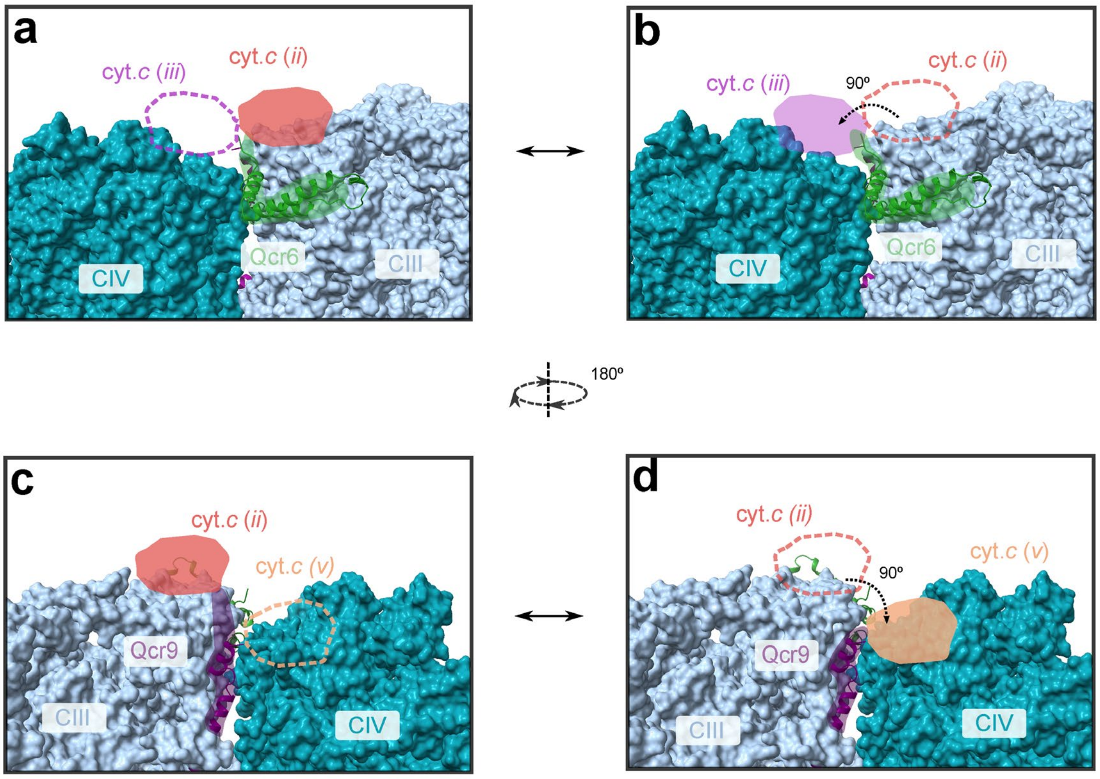

Supplementary Figure S8. Schematic illustration of cyt. *c* interactions with Qcr6/9 while bound at CIII$_2$/CIV. (**a**) Cyt. *c* bound at a CIII monomer of CIII$_2$ with interacting Qcr6 (cyt. *c* position (*ii*), see **Figure** Error! Bookmark not defined.). Cyt. *c* at CIV (position (*v*), see **Figure** Error! Bookmark not defined.) is also shown (dashed). (**b**) Cyt. *c* bound at CIV (position (*iii*)) with interacting Qcr6, based on data from [8]. If Qcr6 would remain bound to cyt. *c* during transition from (*ii*) to (*iii*) cyt. *c* would rotate by ~90° as indicated by the arrow. The proposed rotation of cyt. *c* is based on a comparison of the crystal structures of the cyt. *c*-CIII$_2$ co-complex from *S. cerevisiae* [4] and the cyt. *c*-CIV co-complex from bovine heart mitochondria [5] (see **supplementary Figure S7**). (**c**, **d**) Cyt. *c* bound at a CIII monomer of CIII$_2$ (**c**) (position (*ii*)) or at CIV (position (*v*)) (**d**), interacting with Qcr9. Note the 180° rotation of the views in panels (**a**)/(**b**) and (**c**)/(**d**), respectively.

Table S1. Cryo-EM data collection, refinement and validation statistics

**Data collection and processing**	$CIII_2CIV_1$
Magnification	105,000
Voltage (kV)	300
Electron exposure ($e^-/Å^2$)	40
Defocus range (μm)	0.6 – 2.0
Pixel size (Å)	0.8464
Symmetry imposed	None (C1)
Initial particle images (no.)	2,252,229
Final particle images (no.)	196,457
Map resolution (Å)	2.4
FSC threshold	0.143
Map resolution range (Å)	2.3 – 8.9
**Refinement**	
Initial model used (PDB code)	6HU9
Model resolution (Å)	2.7
FSC threshold	0.5
Map-sharpening *B* factor ($Å^2$)	61.3
Model composition	
Nonhydrogen atoms	48,795
Protein residues	5,846
Ligands	60
B factors ($Å^2$)	
Protein	62.19
Ligand	88.38
Root mean square deviations	
Bond lengths (Å)	0.005
Bond angles (°)	0.614
Validation	
MolProbity score	1.71
Clashscore	4.62
Poor rotamers (%)	0.22
Ramachandran plot	
Favored (%)	96.87
Allowed (%)	3.13
Disallowed (%)	0.00

References

1 Van Gelder, B. F. & Slater, E. C. The extinction coefficient of cytochrome c. *BBA - Biochimica et Biophysica Acta* **58**, 593-595 (1962).

2 Vanneste, W. H. The stoichiometry and absorption spectra of components a and a-3 in cytochrome c oxidase. *Biochemistry* **5**, 838-848 (1966).

3 Varadi, M. *et al.* AlphaFold Protein Structure Database: massively expanding the structural coverage of protein-sequence space with high-accuracy models. *Nucleic Acids Research* **50**, D439-D444, (2021).

4 Lange, C. & Hunte, C. Crystal structure of the yeast cytochrome bc_1 complex with its bound substrate cytochrome *c*. *Proc. Natl. Acad. Sci. USA* **99**, 2800-2805, (2002).

5 Shimada, S. *et al.* Complex structure of cytochrome *c*–cytochrome *c* oxidase reveals a novel protein–protein interaction mode. *EMBO J.* **36**, 291-300, (2017).

6 Scharlau, M. *et al.* Definition of the Interaction Domain and Electron Transfer Route between Cytochrome c and Cytochrome Oxidase. *Biochemistry* **58**, 4125-4135, (2019).

7 Millett, F., Havens, J., Rajagukguk, S. & Durham, B. Design and use of photoactive ruthenium complexes to study electron transfer within cytochrome bc_1 and from cytochrome bc_1 to cytochrome *c*. *Biochim. Biophys. Acta* **1827**, 1309-1319, (2013).

8 Moe, A., Trani, J. D., Rubinstein, J. L. & Brzezinski, P. Cryo-EM structure and kinetics reveal electron transfer by 2D diffusion of cytochrome *c* in the yeast III-IV respiratory supercomplex. *Proc. Natl. Acad. Sci. USA* **118**, e2021157118, 2021157111-2021157119, (2021).
